# Optic Nerve Sheath Diameter as a Bedside Assessment for Elevated Intracranial Pressure

**DOI:** 10.1155/2017/3978934

**Published:** 2017-04-05

**Authors:** Peter Williams

**Affiliations:** Western Health, Gordon Street, Footscray, VIC 3011, Australia

## Abstract

A previously fit and healthy 26-year-old lady with no significant medical history presented with a two-month history of headaches. The headaches were prolonged, generalised, and unusually severe for the patient. Examination revealed papilloedema. The patient's optic nerve sheath diameter was measured 3 mm posterior to the globe and found to be 7.5 mm. The patient subsequently had computed tomography scan of her brain that showed an optic nerve sheath diameter of 7.56 mm as measured 3 mm posterior to the globe. After an obstructive lesion was ruled out on the computed tomography scan, a lumbar puncture was then performed and cerebrospinal fluid was drained. An ultrasound of the optic nerve sheath diameter was repeated showing a reduced diameter of 5.6 mm. The patient was admitted to the neurology unit and ultimately diagnosed with idiopathic intracranial hypertension. This case report highlights the potential of rapidly identifying elevated intracranial pressure using a noninvasive method.

## 1. Introduction

Elevated intracranial pressure (EICP) is common complication of neurological disorders and head trauma. EICP is frequently managed in the Intensive Care Unit (ICU). Invasive intracranial pressure monitoring requires a craniotomy. Ultrasound is emerging as a noninvasive tool that can reliably detect EICP by measuring the diameter of the optic nerve sheath.

## 2. Case Presentation

A previously fit and healthy 26-year-old lady with no significant medical history presented with a two-month history of headaches. The headaches were prolonged, generalised, and unusually severe for the patient. Examination revealed papilloedema.

**Figure 1:**
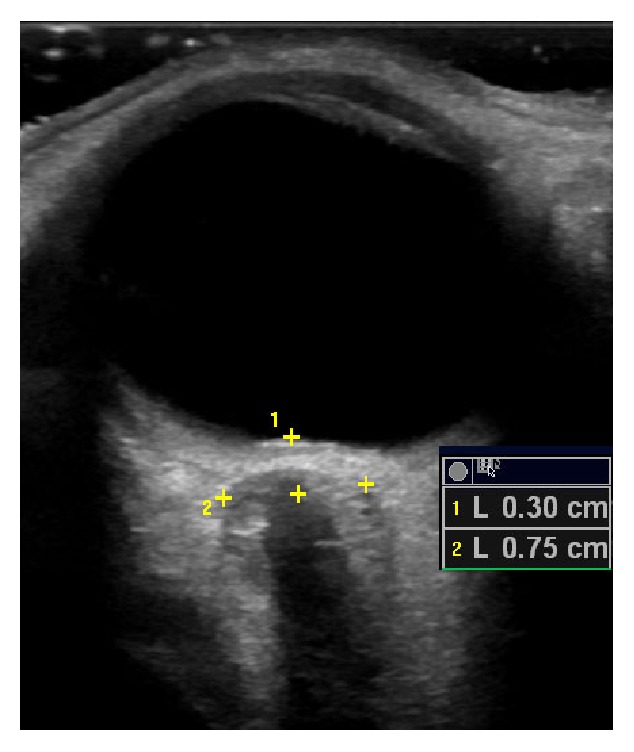
Showing an ultrasound scan with an ONSD of 7.5 mm measured 3 mm posterior to the globe.

**Figure 2:**
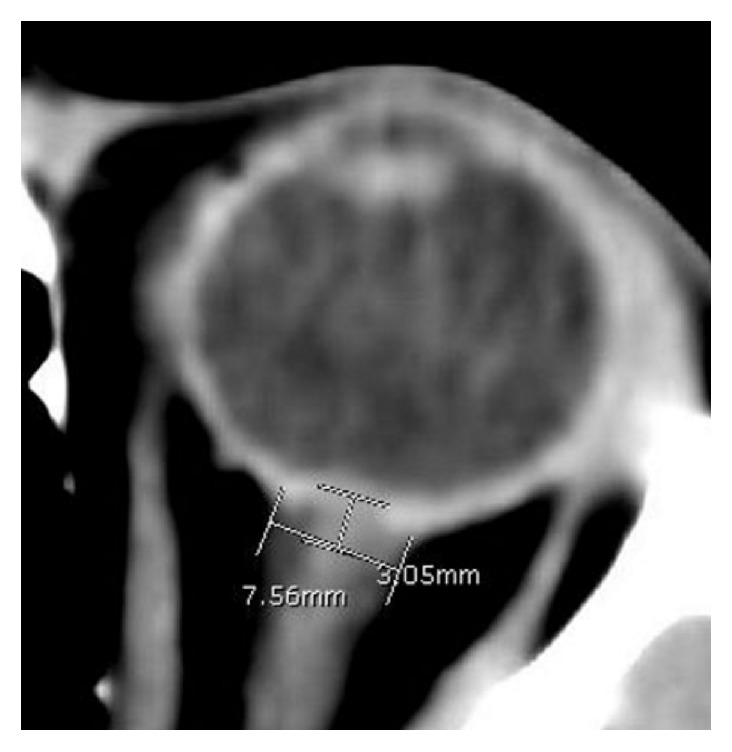
Showing a CT scan with an ONSD of 7.56 mm measured 3 mm posterior to the globe.

The patient was scheduled to have a computed tomography (CT) scan of her brain. Just prior to the CT an ultrasound of her left and right eye was performed to ascertain the optic nerve sheath diameter (ONSD). Measurements were made 3 mm posterior to the point at which the optic nerve enters the globe. The ONSD was 7.5 mm on the ultrasound (see Figure 1). The CT showed an ONSD of 7.56 and revealed no obstructive lesions (see Figure 2). The CT revealed features of an elevated intracranial pressure (EICP).

Subsequently the patient had a lumbar puncture (LP) in a lateral decubitus position. The opening pressure was greater than 35 cm H_2_O and the closing pressure was 20.5 cm H_2_O.

An ultrasound was repeated after the LP. The ONSD was now 5.6 mm. The patient was admitted under the neurology unit and ultimately diagnosed with idiopathic intracranial hypertension (IIH).

## 3. Discussion

The optic nerve is part of the central nervous system and is surrounded by cerebrospinal fluid (CSF) encased in a sheath, the optic nerve sheath. This sheath is continuous with the dura mater and diameter of this sheath changes rapidly with changing CSF pressure.

An increasing evidence base suggests that ultrasound of the OSND is a simple and sensitive way of assessing for the presence of EICP. Primary research by Hansen and Helmke showed a correlation between rising cerebrospinal fluid (CSF) pressure and ONSD [1]. In an emergency department (ED) setting, Blaivas et al. suggested that a cutoff value of 5 mm for the ONSD is sensitive for CT findings consistent with EICP [2]. Compared to invasive monitoring of ICP, various studies have identified a cutoff between 4.8 mm and 5.9 mm for ONSD correlation with EICP [2–9]. Moretti et al. [3] compared ultrasound measurement of the ONSD to invasive ICP monitoring and found an ONSD cutoff of 5.2 mm to be 93% sensitive and 74% specific for EICP. Rajajee et al. [9] found an ONSD cutoff of 4.8 mm to be 96% sensitive and 94% specific for EICP.

CT is readily available to most Intensive Care Units (ICU) in Australia. Nevertheless a noninvasive method to detect EICP would be useful as it may lead to a faster diagnosis and avoid potential problems with transporting ICU patients.

Assessments of ONSD with ultrasound are operator dependent. Assessments are subject to errors of measurement including those relating to artefact, shadowing, and foreshortening. However, ultrasound of the ONSD is easier to learn than other beside assessments of ICP including transcranial Doppler and ophthalmoscopy [10].

This case report highlights the potential of rapidly identifying EICP using a noninvasive method. Ultrasound of the ONSD is a potentially useful way of ruling out EICP in a critical care setting.
